# Platelet mitochondrial membrane depolarization reflects disease severity in patients with preeclampsia

**DOI:** 10.1186/s10020-022-00472-x

**Published:** 2022-05-04

**Authors:** Bjoern F. Kraemer, Irina Hennis, Anne Karge, Anne Katrin Kraemer, Tobias F. Dreyer, Marion Kiechle, Bettina Kuschel, Holger Bronger

**Affiliations:** 1 Medizinische Klinik Und Poliklinik I, LMU Klinikum, Munich, Germany; 2 Department of Gynecology and Obstetrics, Technical University of Munich, Ismaninger Str. 22, 81675 Munich, Germany

**Keywords:** Preeclampsia, Platelets, Mitochondrial membrane potential

## Abstract

**Background:**

Thrombocytopenia is a feared complication of preeclampsia (PE) that can additionally complicate the disease course and that carries a poor prognosis. The disease mechanisms of PE on a platelet level are poorly understood and only few platelet-based markers have been investigated. In sepsis, platelet mitochondrial membrane depolarization, a sensitive and early indicator of mitochondrial dysfunction and platelet cell death, correlates with disease severity and outcome as shown in previous studies. The aim of this study was to investigate platelet mitochondrial membrane potential (Mmp-Index) by flow-cytometry in patients with preeclampsia compared to controls and to assess its value in correlation with disease severity of PE and during follow-up after delivery.

**Methods:**

In this prospective translational case–control study, platelet Mmp-Index was measured in PE (n = 16) by flow cytometry in living platelets in simultaneous comparison to healthy pregnant (n = 32) and non-pregnant controls (n = 16) and was individually reassessed after delivery to investigate recovery of platelet mitochondrial function. Subgroup analysis of patients with severe and non-severe PE was performed. Six patients with isolated gestational hypertension were also included for comparative analysis.

**Results:**

Platelet Mmp-Index in patients with symptomatic preeclampsia (Mmp-Index non-severe PE 0.72 ([0.591; 0.861]; p = 0.002) was significantly reduced compared to healthy pregnant controls (Mmp-Index 0.97 [0.795; 1.117]) and even more pronounced in patients with severe PE (n = 6) (Mmp-Index severe PE 0.542 [0.361; 0.623]; p = 0.03). In the severe PE group, complementary measurements of platelet Annexin V- and CD62 (P-Selectin) surface expression showed apoptosis of platelet populations in the majority of patients. Platelet Mmp normalized after delivery within few days. Patients with isolated gestational hypertension showed normal Mmp-Index values.

**Conclusions:**

This study shows for the first time that platelet Mmp-Index is a quantifiable, easy-to-measure intracellular marker of platelet mitochondrial function in vital cells that reflects disease severity of preeclampsia. For future investigations, platelet Mmp may serve as a prognostic marker that may aid clinical risk stratification and adds novel information on potential mechanisms for thrombocytopenia in preeclampsia.

**Supplementary Information:**

The online version contains supplementary material available at 10.1186/s10020-022-00472-x.

## Background

Preeclampsia (PE) is a leading cause of morbidity and mortality for mother and fetus that is estimated to occur in about 5% of pregnancies (Abalos et al. 2013). Preeclampsia is characterized by hypertension and proteinuria in pregnancy that can progress to severe organ damage, frequently including thrombocytopenia (ACOG practice bulletin). Besides this classic clinical definition, PE can also present in the absence of proteinuria as shown in the same guideline. Steady progress has been made to elucidate disease mechanisms on a placental level and molecular markers such as the sFlt-1/PIGF ratio (soluble Fms-like Tyrosinkinase-1/Placental growth factor) (Levine et al. 2004; Zeisler et al. 2016) have improved prediction for occurrence of preeclampsia. The complex disease pathology of PE involves systemic inflammation mediated by: dysregulated trophoblast invasion and angiogenesis (Whitley et al. 2009), placental ischemia, inflammatory microparticle release (Cheng et al., 2019), endothelial and neutrophil-activation (Major et al. 2014; Terrone et al. 2000) that lead to vascular injury and multi-organ failure (Cheng et al. 2019; Stojanovska et al. 2020). Thrombocytopenia is another characteristic finding in PE and is even an independent factor that defines severe cases of PE according to guideline definitions, although it is unclear why especially platelets are affected. Several protein markers from plasma (Grill et al. 2009) or urine (Stojanovska et al. 2020) are currently under investigation as adjunct parameters for disease severity in PE. However, with regard to platelet metabolism, only few studies have been conducted so far (Malinow et al. 2018; Kramer et al. 2014; Pimentel et al. 2013; Zemel et al. 1990), while other studies demonstrated changes in platelet cell volume (Thalor et al. 2019; Piazze et al. 2006), increased platelet-monocyte co-aggregate formation (Major et al. 2014) and upregulation of P-Selectin on platelets from patients with PE (Holthe et al. 2004; Konijnenberg et al. 1997; Yoneyama et al. 2001). Earlier studies have shown that thrombocytopenia is also an indicator of poor clinical outcome in sepsis (Akca et al. 2002; Strauss et al. 2002; Vandijck et al. 2010). As previously demonstrated, thrombocytopenia is not a random phenomenon, but platelet lifespan is determined by a regulated apoptotic mechanism that eventually leads to mitochondrial dysfunction and cell death (Kile 2009; Mason et al. 2007). Breakdown of mitochondrial membrane potential is an early indicator for mitochondrial compromise in the intrinsic cell death pathway of platelets (Leytin et al. 2009) which can be activated during sepsis (Kraemer et al. 2012). In previous work, we and others were able to demonstrate that platelet mitochondrial membrane depolarization in platelets from sepsis patients directly correlates with the disease severity of sepsis (Grundler et al. 2014; Yamakawa et al. 2009) and outcome (Grundler et al. 2014). Investigations on a placental level also show mitochondrial damage and increased mitochondrial DNA release as pathomechanisms of PE (Smith et al. 2021; Vangrieken et al. 2021). Platelet cell death pathways can be activated independently of platelet activation and meticulous investigations show that platelet apoptosis and activation are separate processes (Gyulkhandanyan et al. 2013; Leytin et al. 2008). Platelets thus appear to be interesting targets for cell markers that potentially predict and reflect disease course of PE since platelets are blood cells that are typically directly affected as part of the disease phenotype and in the pathophysiology of PE. Because mechanisms that lead to thrombocytopenia in preeclampsia are basically unknown, we conducted this prospective case–control study to measure platelet Mmp by flow cytometry in living platelets from patients with symptomatic preeclampsia before and after delivery for the first time.

The aim of this study was to investigate if platelet mitochondrial membrane potential quantified by Mmp-Index is affected in preeclampsia, if there are differences depending on disease severity and how the effects change or are reversible after delivery. The investigation further aims to provide the experimental basis to get a better understanding of potential disease mechanisms of preeclampsia on a platelet level and to find out if platelet Mmp could eventually serve as a platelet-based cellular marker of disease activity in preeclampsia.

## Materials and methods

### Study population

Sixteen Patients were admitted with the diagnosis of PE according to the ACOG guidelines (ACOG Practice Bulletin 2019) as well as 32 normotensive, healthy pregnant controls (16 patients each for antepartum/postpartum control groups) and 16 healthy non-pregnant controls were prospectively included in the study between July 2020 and March 2021. Prior sample size calculation, assuming a 30% relative reduction in Mmp-Index in PE resulted in a sample size estimate of 13 patients per group. Six Patients with isolated gestational hypertension but no additional signs of preeclampsia or manifestation of organ damage such as proteinuria, elevated liver enzymes, renal failure or thrombocytopenia were included as a separate group for analysis. Women with preeclampsia or control patients on anti-aggregatory platelet therapy or drugs that affect platelet function, with preexisting platelet or coagulation disorders, diabetes, renal disease as well as patients with signs of infection or preexisting disease of liver or bone marrow were excluded from the study. Blood was drawn simultaneously from PE and control patients. The patient collective comprised 16 patients with PE, six of which suffered from severe PE, which was in this study defined as the presence of at least two “severe” ACOG guideline criteria. These criteria are: hypertension > 160 mmHg systolic or 110 mmHg diastolic, thrombocytopenia < 100.000/µl, impaired liver function, renal failure, pulmonary edema or neurological deficits. Blood and urine analysis for CBC, liver enzymes, kidney function, hemolysis and proteinuria were performed on admission. Blood was immediately transferred to the lab for analysis of platelet Mmp by flow-cytometry. After delivery, follow-up blood draws were taken on average after 2.2 days (median 2 days; range 1–4 days). Follow-up blood draws were also compared to a simultaneous healthy pregnant and non-pregnant control sample. The study was approved by the local ethics committee of the Technical University of Munich in accordance with the Declaration of Helsinki (Approval 104/20S-KH). Written informed consent was obtained from all patients and study participants prior to blood sampling.

### Platelet isolation

Platelets were isolated from whole blood as previously described (Grundler et al. 2014). Accordingly, whole blood from patients with PE and from healthy pregnant and non-pregnant control patients was drawn directly into commercially available plastic tubes containing sodium citrate (1:10). After centrifugation of the whole blood without brake at 340×*g* for 15 min at room temperature (RT), the platelet -rich plasma (PRP) was carefully removed and given to preheated platelet isolation buffer (138 mM NaCl, 2.7 mM KCl, 12 mM NaHCO3, 0.4 mM NaH_2_PO_4_, 1 mM MgCl, 5 mM d-Glucose and 5 mM HEPES). Prior to centrifugation, Prostaglandin E1 (1 µM, Santa Cruz Biosciences) was added to prevent platelet activation and platelets were pelleted at 600×*g* for 10 min at room temperature. Platelet poor plasma (PPP) was carefully removed and isolated platelets were resuspended at equal concentrations in platelet isolation buffer for further use.

### Measurement of mitochondrial membrane potential by flow cytometry

Mitochondrial membrane potential (Mmp) was measured with JC-1 dye by flow cytometry, according to the manufacturer’s instructions (Immunochemistry Technologies, Bloomington, MN, USA). Purification and resuspension of platelets at equal concentrations guaranteed comparable staining and processing conditions. Samples from patients with thrombocytopenia were thus comparable to samples with normal platelet numbers. JC-1 fluorescent dye was incubated with platelets for 20 min at RT in the dark. Platelet isolation buffer was added afterwards (1 ml) for immediate use of cells by flow cytometry. Flow cytometry was performed by using a BD Calibur flow cytometer (BD Biosciences, Heidelberg, Germany). Mitochondrial membrane potential (Mmp) was assessed as a ratio of the mean FL2 (red fluorescence) and FL1 (green fluorescence) (Grundler et al. 2014). We refer to this ratio as “Mmp-Index” in the text. A decrease in the FL2-to-FL1 ratio (Mmp-Index) represents a loss in mitochondrial membrane potential (depolarization). Mmp-Index was calculated as the mean of triplicate readings. Stimulation with calcium ionophore A23187 (Calbiochem, Darmstadt, Germany) at 10 μM for 10 min, which induces rapid mitochondrial membrane depolarization, was used as an internal positive control to assure correct function of the assay.

### Flow cytometry for Annexin V and P-Selectin (CD62P) surface expression

Detection of Phosphatidylserine (PS) surface expression on platelets from PE patients and corresponding pregnant controls by Annexin V staining was performed by flow cytometry using an Annexin V expression Kit (FITS) according to the manufacturer`s instructions (BD Biosciences, Germany). For CD62 (P-Selectin) surface expression, isolated platelets from PE and control samples were incubated with a PE-labeled anti-CD62 (P-Selectin) antibody and control IgG for 15 min in the dark at RT (BD Biosciences, Germany). Afterwards cells were washed in sterile PBS, centrifugated at 450×*g* for 10 min without brake and gently resuspended in sterile PBS for immediate flow cytometry analysis. Flow cytometry readings were done using a BD Calibur flow cytometer (BD Biosciences, Heidelberg, Germany).

### Statistical analysis

Analyses of Mmp data and patient characteristics were performed using Wilcoxon matched-pairs signed rank test or Mann Whitney test. All tests were performed on exploratory two-sided 5% levels of significance (GraphPad Prism, Version 8.0.1, Graphpad Software, Inc.).

## Results

### Patient characteristics

Sixteen patients admitted with preeclampsia, 32 healthy pregnant controls (16 patients each for antepartum/postpartum control groups) with normal blood pressure as well as 16 non-pregnant controls were included in the study. Baseline patient characteristics are shown in Table 1 and Additional file 3: Table S1. Three patients in the PE group did not exceed cut-off values for proteinuria, but were included in the PE group due to elevated liver enzymes (ACOG Practice Bulletin 2019). Median age of patients with PE was 32 [30; 35] years compared to 34 [30; 38] years in the healthy pregnant group and did not statistically differ (p = 0.49). Gestational age was lower in the PE group 35 [32; 37] weeks at the time of analysis versus 39 [36; 39] weeks in healthy control patients (p = 0.0029). There was no correlation between Mmp-Index and age or gestational age in either group (data not shown). Blood pressure measurements in the PE group were significantly higher (median systolic RR 154 mmHg versus 119 mmHg, median diastolic 99 mmHg versus 75 mmHg; p < 0.0001). Median and interquartile range of sFlt-1/PIGF ratios in the PE group was 143 [111; 240] and there was no correlation to Mmp-Index values (data not shown). Among patients with PE, six patients suffered from severe preeclampsia according to guideline definitions (ACOG Practice Bulletin 2019). Severe PE criteria are: blood pressure > 160 mmHg systolic or 110 mmHg diastolic, thrombocytopenia (< 100.000/µl), elevated liver encymes, renal failure, pulmonary edema or neurologic symptoms. Slightly modified in this study, patients were only classified as “severe preeclampsia” when they fulfilled at least two “severe PE” guideline criteria. Two patients with severe PE did not exceed cut-off values for hypertension, due to signs of beginning shock and one patient was included based on clinical signs with new-onset severe headache and neurological deficits. All other patients were included in the “non-severe PE”- group. Only one of the PE patients showed platelet counts of < 100.000/µl at the time of blood sampling. Platelet counts were lower in the PE group compared to pregnant controls but did not reach statistical significance (154 [125; 183] versus 187 [154; 205]; p = 0.11). No patient received aspirin or other anti-aggregatory or anti-coagulant therapy at the time of blood sampling to exclude drug-dependent effects. Six patients with isolated gestational hypertension (median systolic 159 mmHg/diastolic 93 mmHg) were analyzed outside the primary study group for comparison. Patients with isolated gestational hypertension showed normal platelet levels. Average time of follow-up blood draw after delivery was 2.2 days (median 2 days; range 1–4 days).

**Table 1:** Patient baseline characteristics

Group	Preeclampsia	Pregnant control	Non-pregnant control	p-value	Gestational HTN
No. of patients (n)	**16**	**16**	**16**		**6**
Age [years]	32 [30; 35]	34 [30; 38]	24 [24; 26]	0.49	36 [33; 38]
Gestational age [weeks]	35 [32; 37]	39 [36; 39]		0.0029	37 [36; 38]
Blood pressure systolic (mmHg)	154 [149; 171]	119 [113; 124]		< 0.0001	159 [154; 168]
Blood pressure diastolic (mmHg)	99 [90; 113]	75 [71; 77]		< 0.0001	93 [91; 99]
Platelet count [*1000/µl]	154 [125; 183]	187 [154; 205]		0.11	172 [162; 175]

Values show median and [interquartile range]. Pregnant control represents the 16 controls for the PE antepartum group

### Platelet mitochondrial membrane potential is significantly decreased in patients with preeclampsia

**Fig. 1:**
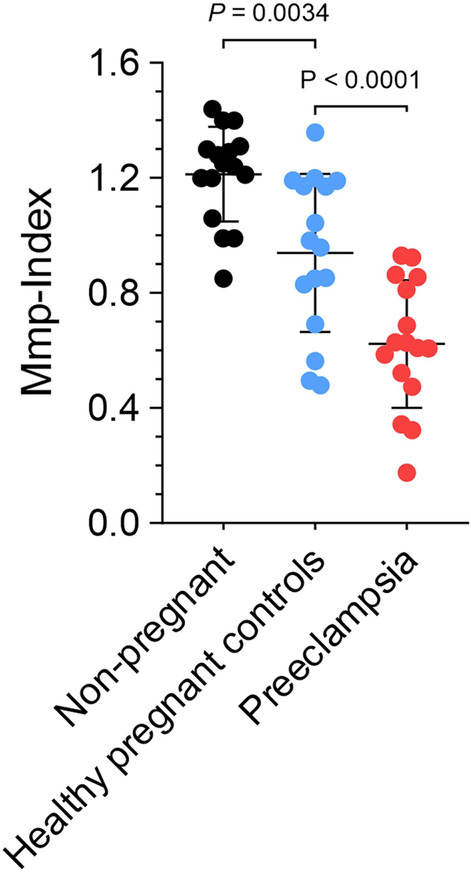
Platelet mitochondrial membrane potential is decreased in preeclampsia. Mmp-Index of patients with preeclampsia (PE) (n = 16), healthy pregnant controls and non-pregnant controls is shown. Mitochondrial membrane potential (Mmp) of platelets from patients with PE is significantly reduced compared to healthy pregnant controls (p < 0.0001). Baseline platelet Mmp-Index of pregnant patients was also significantly lower than in non-pregnant women at baseline (p = 0.0034)

Sixteen patients with PE compared to 16 healthy pregnant and 16 non-pregnant controls were included in the analysis. Matched pair analysis of all patients with PE showed significantly decreased absolute Mmp-Index values of 0.618 [0.510; 0.822] compared to healthy pregnant controls 0.97 [0.795; 1.117] (p < 0.0001; Fig. 1). At baseline, platelet Mmp-Index was also significantly lower in pregnant compared to non-pregnant patients (1.245 [1.165; 1.301]) (p = 0.0034). Additional file 1: Fig. S1 illustrates a flow cytometry analysis of Mmp-Index of a sample triplet comparing PE, pregnant and non-pregnant control.

### Platelet mitochondrial membrane depolarization reflects disease severity of patients with preeclampsia

**Fig. 2:**
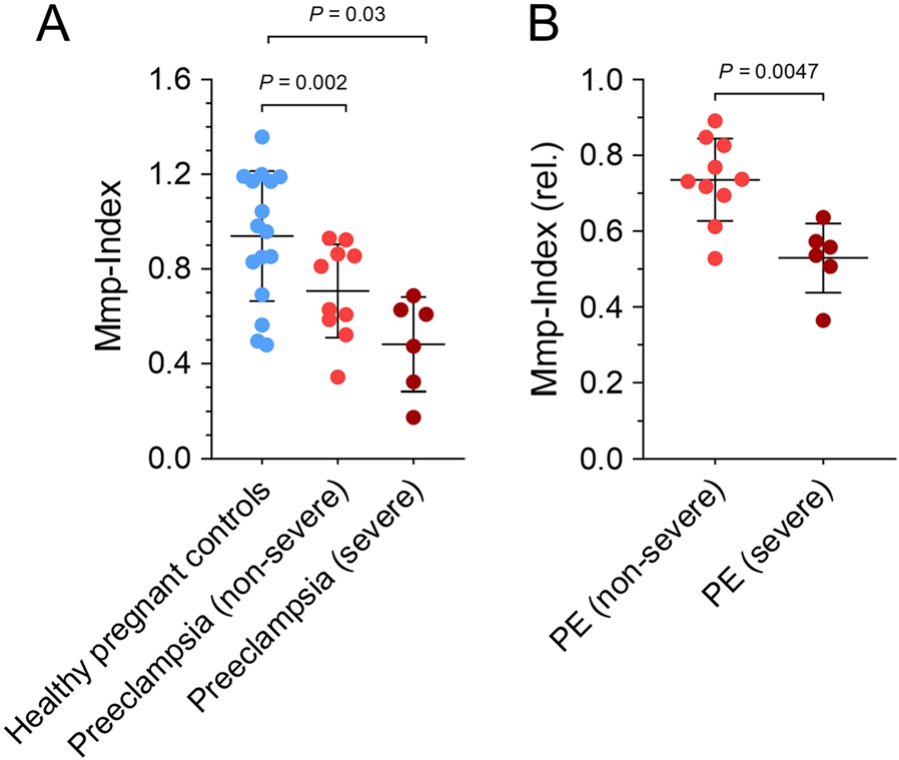
Platelet mitochondrial membrane depolarization reflects the disease severity of patients with preeclampsia**.** **A** Subgroup analysis of PE patients with severe (n = 6) and non-severe (n = 10) PE showed a significantly reduced Mmp-Index compared to pregnant controls (p = 0.03 and p = 0.002). **B** Relative Mmp-Index ratios are significantly lower in severe compared to non-severe preeclampsia (p = 0.0047)

Patients with preeclampsia were subclassified into non-severe (n = 10) and severe PE (n = 6) according to guideline definitions. Both non-severe and especially severe PE patient groups showed significantly reduced absolute platelet Mmp-Index values (non-severe 0.720 [0.591; 0.861]; p = 0.002 and severe 0.542 [0.361; 0.623]; p = 0.03) compared to healthy pregnant controls (Fig. 2A). Figure 2B illustrates the subgroup analysis for PE patients with relative Mmp-Index ratios (ratio of PE compared to their corresponding same-day pregnant control) which shows a significantly lower relative Mmp-Index in severe 0.547 [0.514; 0.569] compared to non-severe PE patients (0.734 [0.701; 0.812] (p = 0.0047).

### Platelet mitochondrial membrane depolarization during PE promptly normalizes within few days after delivery

**Fig. 3:**
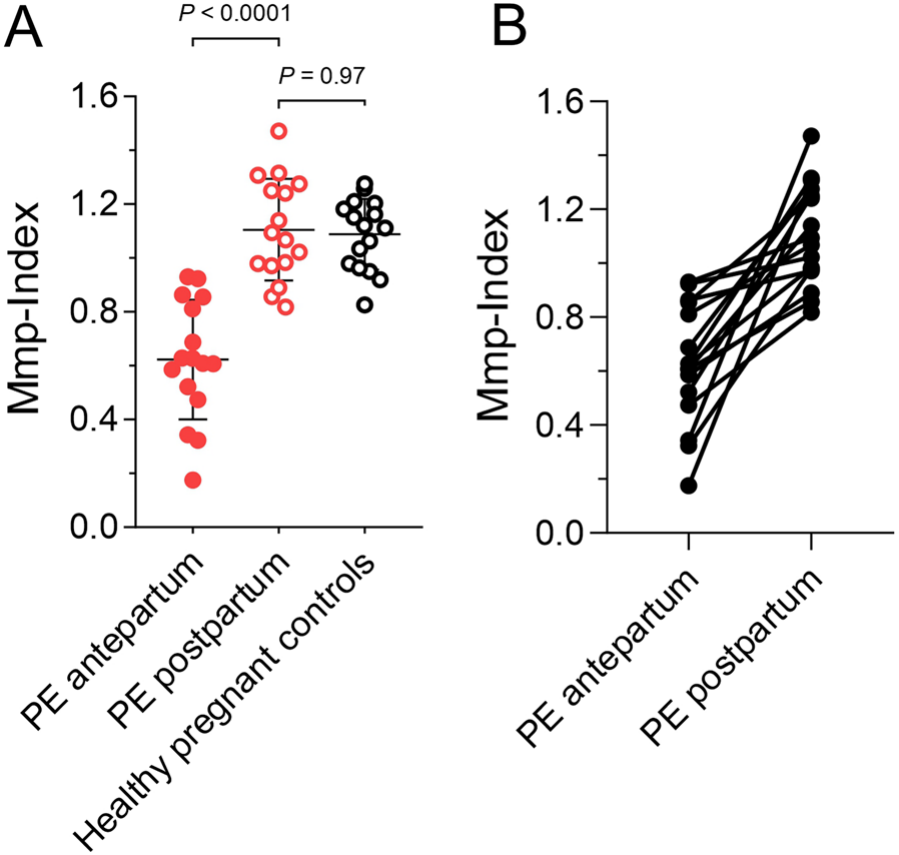
Platelet mitochondrial membrane depolarization during PE normalizes to baseline levels after delivery. **A** Platelet Mmp-Index of patients with PE (PE antepartum) increases after delivery (PE postpartum) and normalizes to baseline levels of normal pregnant controls (p = 0.97). **B** Illustration of the individual development of platelet Mmp-Index for each PE patient pre- (antepartum) and post-delivery

Platelet Mmp-Index values of patients with PE 0.618 [0.510; 0.822] (PE antepartum) significantly increased and normalized within few days after delivery to 1.081 [0.978; 1.256] (PE postpartum) (p < 0.0001). After delivery, absolute platelet Mmp-Index values of former PE patients did no longer differ from normal pregnant controls (1.115 [0.976; 1.187], p = 0.97) (Fig. 3A) and healthy non-pregnant controls 1.245 [1.165; 1.302], p = 0.188). Figure 3B illustrates the individual recovery of platelet Mmp-Index for each patient with preeclampsia before and after delivery.

**Fig. 4:**
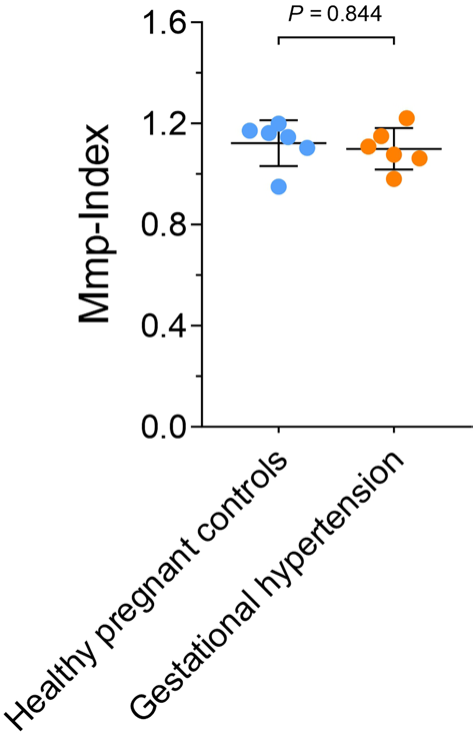
Comparison of platelet Mmp-Index between patients with isolated gestational hypertension and normal pregnancy. Platelet Mmp-Index of patients with isolated gestational hypertension (n = 6) in the absence of preeclampsia showed no statistical difference to pregnant control patients (p = 0.844)

### Platelet mitochondrial membrane potential is not affected in patients with isolated gestational hypertension in the absence of PE

Patients with isolated gestational hypertension (n = 6), without preeclampsia showed no statistical difference in Mmp-Index compared to the corresponding pregnant controls (1.093 [1.066; 1.139] versus 1.154 [1.114; 1.170]; p = 0.844) (Fig. 4).

### Platelets predominantly show an apoptotic phenotype in severe PE

Based on the molecular definition of apoptotic platelets by comprehensive work of Leytin and colleagues (Gyulkhandanyan et al. 2013; Leytin et al. 2008), platelets show an apoptotic molecular phenotype in case of Mmp depolarization (or in late apoptotic stages Annexin V surface expression) (Reddy et al. 2020) in the absence of P-selectin surface overexpression (Annexin V (+)/CD62 (−)). Mixed cases of apoptosis and activation show both Annexin V and P-Selectin upregulation (Annexin V (+)/CD62 (+)) while Mmp depolarization without Annexin V and CD62 surface overexpression (Annexin V (−)/CD62 (−)) is most likely an early, reversible proapoptotic state. We acquired Annexin V and P-Selectin surface expression data from 13 of our patients with preeclampsia in direct comparison to their corresponding pregnant controls, while we lack data for three patients from the non-severe PE group. Our analysis shows apoptotic platelet populations (Annexin V (+)/CD62 (−)) in the majority (4 out of 6) of patients from the severe PE group and in one patient from the non-severe PE group. Additional file 2: Fig. S2A illustrates an example of the apoptotic molecular platelet phenotype by flow cytometry. Two patients in the severe PE group and two patients in the non-severe PE group show a mixed phenotype of apoptosis and activation (Annexin V (+)/CD62 (+)) besides mitochondrial membrane depolarization. The remaining four non-severe PE patients only showed Mmp depolarization (Annexin V (−)/CD62 (−)). We have indicated surface expression of Annexin V or CD62 (P-Selectin) for each PE patient compared to their corresponding pregnant control in Additional file 3: Table S1 and have included a four square matrix for visualization of the results as Additional file 2: Fig. S2B.

## Discussion

The aim of this study was to investigate platelet mitochondrial membrane depolarization, a sensitive marker of mitochondrial distress and an early marker of apoptosis, in patients with PE compared to healthy pregnant and non-pregnant controls. We demonstrate for the first time that platelet mitochondrial membrane potential (Mmp-Index) is significantly decreased in patients with PE. Moreover, subgroup analysis revealed that platelet Mmp depolarization reflects disease severity of PE and was unaffected in isolated gestational hypertension. Mitochondrial membrane potential (Mmp-Index) of platelets recovered to baseline levels of healthy pregnant and non-pregnant controls within few days after delivery, which indicates that the gestational environment of PE is indeed responsible for the mitochondrial stress response in platelets.

Diagnostic advancements such as the discovery of the sFlt-1/PIFG ratio (Levine et al. 2004; Zeisler et al. 2016) have significantly improved risk prediction for PE. Other markers (e.g. SERPINA1) measured in urine samples (Starodubtseva et al. 2020) are currently under investigation and several general serum markers such as D-Dimers, fibrinogen, APT or LDH have shown correlation (Duan et al. 2019) with the diagnosis or severity of PE. However, only few studies have focused on the integrity and function of platelets. In particular, mitochondria-based markers for platelet metabolism in vital cells have not been investigated in PE so far. With regard to platelets, previous research has shown that platelet volume (MPV) and shape changes seem to correlate with disease severity and the development of PE (Thalor et al. 2019; Hutt et al. 1994; Mayer-Pickel et al. 2019). However, diverging results (Altinbas et al. 2012) have been reported in other studies, which were interpreted as differences or difficulties in optimal diagnostic timing (Bellos et al. 2018). Work by Mayor and colleagues observed a potentially direct involvement of platelets in the disease process of PE by formation of platelet-monocyte aggregates as a source of sFlt-1 (Major et al. 2014). Other clinical investigations show increased calcium flux (Zemel et al. 1990), procoagulant activity and activation of platelets (P-Selectin) in PE (Holthe et al. 2004; Konijnenberg et al. 1997; Yoneyama et al. 2001). Thrombocytopenia has been associated with a detrimental disease course in patients with sepsis (Vandijck et al. 2010) and preeclampsia (Sitotaw et al. 2018). Previous work from our group and others laid the experimental basis for this study by demonstrating that platelet mitochondrial membrane depolarization correlates with disease severity (Grundler et al. 2014; Yamakawa et al. 2013) and outcome of patients with sepsis (Grundler et al. 2014). The important discovery of platelet apoptosis as a molecular clock mechanism that regulates platelet lifespan (Kile et al. 2009; Mason et al. 2007) has changed the view on the etiology of thrombocytopenia entirely. Inflammatory environments such as sepsis can activate the cell death mechanism in platelets and accelerate platelet apoptosis through an apoptotic pathway that is initiated by breakdown of mitochondrial membrane potential (Gyulhandanyan et al. 2013). Decrease of mitochondrial membrane potential, as shown in our study, can progress to calpain and caspase activation, cytoskeletal fragmentation and upregulation of phagocytosis signals such as Annexin V (Kile 2009; Kile et al. 2014). Likely pro-inflammatory microparticles (Cheng et al. 2019) and reactive oxygen species (Williamson et al. 2017) mediate the systemic stress response that affects secondary organs and platelets.

In our study, we were able to demonstrate for the first time that in living platelets, mitochondrial membrane potential is significantly decreased in PE. Moreover, subgroup analysis of patients with severe manifestation of PE, revealed extensive Mmp depolarization beyond the extend observed in the non-severe PE group. This indicates that the degree of platelet Mmp depolarization seems to reflect disease severity of PE. Supporting these findings, patients with isolated gestational hypertension, but no clinical or laboratory signs of organ dysfunction or PE, showed normal platelet Mmp-Index levels comparable to healthy pregnant controls. Compared to non-pregnant patients however, platelet Mmp-Index of pregnant controls was lower, which is in accordance with previous findings that emphasize a mild inflammatory environment in pregnancy per se (Palm et al. 2013). Baseline characteristics of healthy pregnant controls and PE patients did not differ in an unexpected way between groups, which makes our data and results representative of a real world population. There was no significant difference in platelet counts between the PE group and pregnant controls and there was no correlation between age, gestational age and Mmp-Index in either group. Blood sampling was performed prior to initiation of drug therapy and no patient received anti-aggregatory therapy which excludes systematic study errors due to drug interaction. It is important to note, that in our study, platelet counts in the PE group were mostly still within normal limits, although the reduced mitochondrial membrane potential already indicated mitochondrial distress. This also proves that decrease of Mmp is not simply a reflection of platelet number. In line with this hypothesis, Malinow and colleagues also describe differences in respiratory chain function of platelet mitochondria in pregnant and non-pregnant donors (Malinow et al. 2018) and most importantly, platelets from patients with PE showed a deviation from the normal mitochondrial respiratory reduction of normal pregnancy. Although the results of this different experimental approach that used isolated mitochondria cannot be directly transferred to our findings, this work and other publications (Malinow et al. 2018; Kramer et al. 2014; Pimental et al. 2013) indicate that the placental environment in PE is responsible for the effects on platelet mitochondrial function. We observed a significant recovery of platelet Mmp depolarization to baseline levels of controls within few days (median 2 days) after delivery, which underscores the dynamics and sensitivity of this assay marker. On average platelet Mmp-Index normalized to baseline values within 2 to 3 days, but we observed normalization as early as few hours up to several days post-delivery. More stringent time course measurements will be necessary to investigate the meaning of MMP recovery time in PE in future studies.

An important point of this pioneer study is the establishment of a timely available and quantifiable ex vivo assay that measures platelet mitochondrial membrane potential (Mmp-Index) in living platelets in a prospective study design. An advantage of studying living platelets is the chance to detect mitochondrial malfunction in a cell that is characteristically directly affected by the PE disease phenotype and thrombocytopenia defines severe PE cases. Platelet Mmp depolarization sensitively detects mitochondrial dysfunction and can indicate platelet cell death early before platelet numbers decrease. As mentioned earlier, platelet mitochondrial membrane potential can be reduced as an early sign and stage of the cell death pathway leading to apoptosis. Late markers of apoptosis, downstream of mitochondria in the apoptotic pathway like Annexin V, reflect phosphatidylserine (PS) surface exposure, a phagocytosis signal, on platelets (Leytin et al. 2019; Reddy et al. 2020). Comprehensive investigations by Leytin and colleagues show that platelet apoptosis and activation are distinctly different entities (Gyulkhandanyan et al. 2013) and define that the primary molecular phenotype of apoptosis is indeed characterized by Mmp depolarization in the absence of platelet activation (P-selectin upregulation) (Gyulkhandanyan et al. 2013). Based on this definition, we found populations of platelets with an apoptotic platelet phenotype (Annexin V (+)/CD62 (−)) in the majority of patients with severe preeclampsia and in few patients with non-severe preeclampsia, while four patients showed a mixed phenotype of apoptosis and activation (Annexin V (+)/CD62 (−)). These findings further support the hypothesis that Mmp depolarization seen in PE patients is most likely part of a proapoptotic platelet phenotype and represents an early, reversible state of mitochondrial dysfunction that can lead to complete platelet cell death in PE. This could explain why thrombocytopenia is a typical clinical feature of preeclampsia and even an independent factor that defines severe PE cases. There was no correlation between Mmp-Index and sFlt-1/PIGF in this study, but platelet Mmp-Index could potentially add valuable complementary information about PE disease activity on a platelet level to established diagnostic indicators of preeclampsia such as sFlt-1/PIGF.

Another interesting finding of this study is the rapid and dynamic recovery of platelet Mmp-Index in PE patients after delivery, which underscores that mitochondrial affection seems to be a direct result of the disease activity, environment and pathology of PE on platelets. Most likely the mitochondrial stress response depends on the surrounding inflammatory milieu, as we see minimally decreased platelet Mmp levels already in pregnancy per se, moderate reduction during PE and massive Mmp depolarization during sepsis (Grundler et al. 2014; Yamakawa et al. 2013). Mmp depolarization could thus be a biological sensor for the inflammatory status in PE. It remains to be answered however, what factors in preeclampsia exactly mediate the mitochondrial compromise in platelets. Possible factors could involve cell-derived microparticles, reactive oxygen species, plasma cytokines such as sFlt-1 or cell–cell interaction of platelets with leukocytes or endothelium what will have to be answered in future studies. Larger follow-up studies will also be necessary to determine absolute cut-off values for Mmp-Index for clinical utility.

## Conclusions

In summary, using a quantifiable assay that allows a direct visualization of Mmp in living platelets, we demonstrate for the first time that Mmp depolarization in platelets reflects disease activity in PE. Our data suggest that platelet mitochondrial metabolism is involved in the disease mechanism of thrombocytopenia during preeclampsia which contributes novel aspects to the complex pathophysiology of PE. Our study lays the experimental basis to further investigate and potentially establish platelet Mmp as a biological marker and sensor for disease activity and detrimental disease courses of PE in future studies.

## Supplementary Information

**Additional file 1: Figure S1.** Illustration of JC-1 flow-cytometry analysis of Mmp-Index of a non-pregnant, pregnant, and preeclampsia platelet sample.

**Additional file 2: Figure S2.** A) Representative patient sample pair illustrating the apoptotic molecular phenotype of platelets in PE (Annexin V surface expression without CD62 (P-Selectin) surface overexpression) versus pregnant control. B) 4-square matrix illustration of the number of PE patients showing Annexin V and/or CD62 surface overexpression (total number of patients n=13).

**Additional file 3: Table S1.** Individual clinical baseline characteristics of patients with preeclampsia. **Table S2.** Selected individual baseline characteristics of pregnant controls.

## Data Availability

All data generated or analysed during this study are included in the published article and its supplementary information files.

## Abbreviations

PE: Preeclampsia
CBC: Complete blood count
Mmp: Mitochondrial membrane potential
RT: Room temperature
PBS: Phosphate buffered saline
