# Supplementary figures and images for: Platelet mitochondrial membrane depolarization reflects disease severity in patients with preeclampsia
Source: Mol Med. 2022 May 4;28:51. doi: 10.1186/s10020-022-00472-x (PMC9066965; doi:10.1186/s10020-022-00472-x)

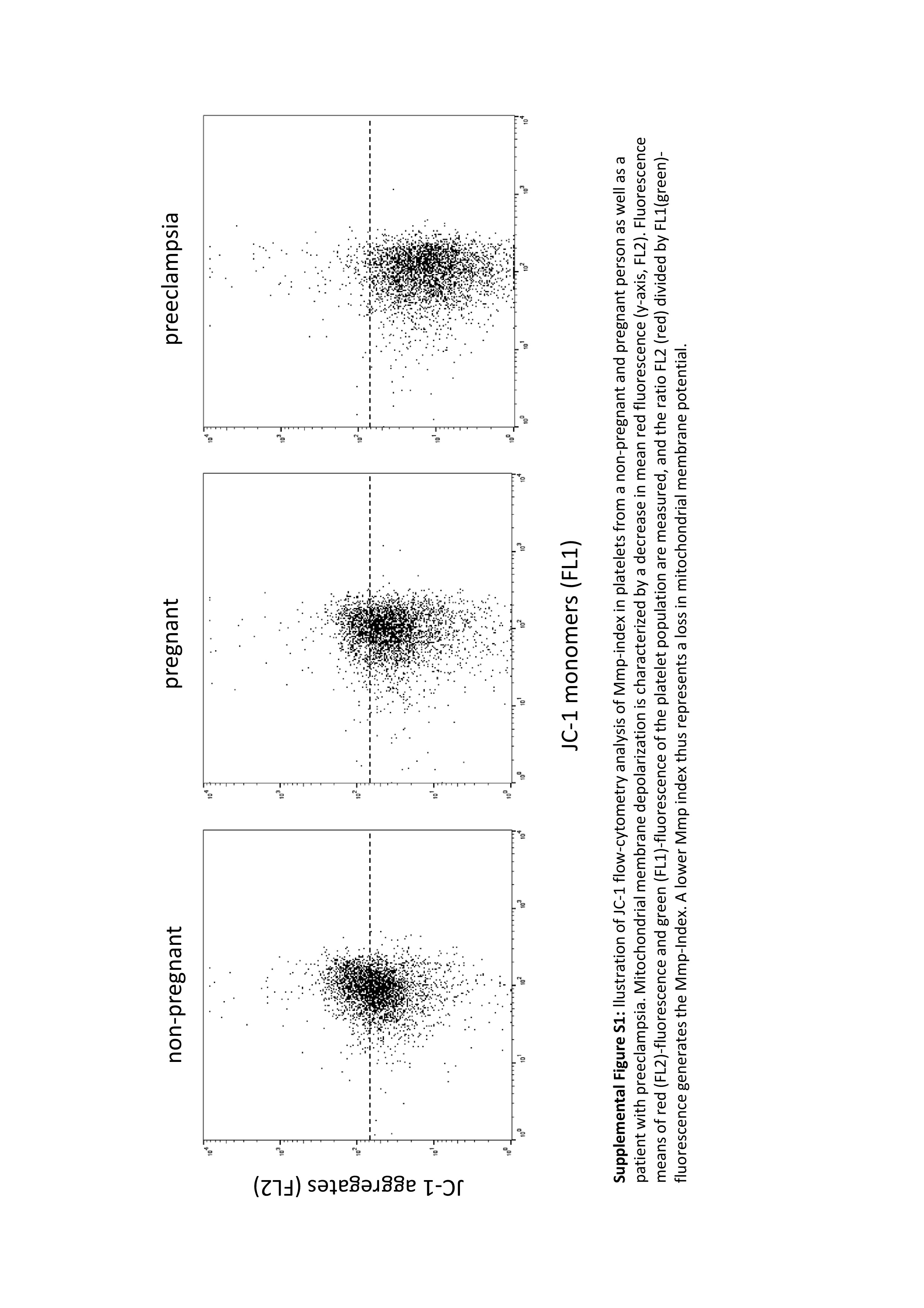

Supplement: Supplementary file 1 — Additional file 1: Figure S1. Illustration of JC-1 flow-cytometry analysis of Mmp-Index of a non-pregnant, pregnant, and preeclampsia platelet sample. [file 10020_2022_472_MOESM1_ESM.jpg]

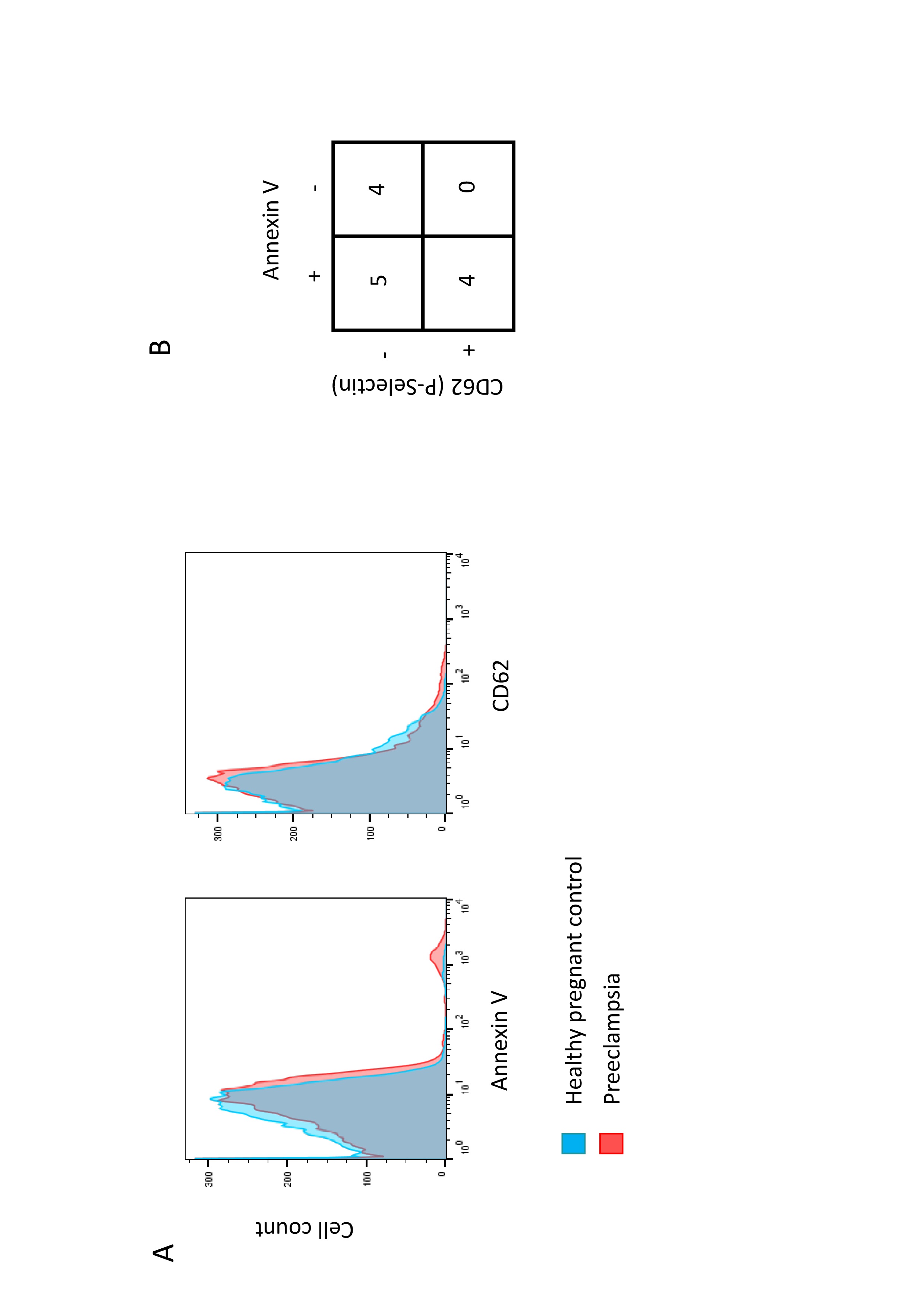

Supplement: Supplementary file 2 — Additional file 2: Figure S2. A) Representative patient sample pair illustrating the apoptotic molecular phenotype of platelets in PE (Annexin V surface expression without CD62 (P-Selectin) surface overexpression) versus pregnant control. B) 4-square matrix illustration of the number of PE patients showing Annexin V and/or CD62 surface overexpression (total number of patients n=13). [file 10020_2022_472_MOESM2_ESM.jpg]
