# Supplementary material for: Platelet mitochondrial membrane depolarization reflects disease severity in patients with preeclampsia
Source: Mol Med. 2022 May 4;28:51. doi: 10.1186/s10020-022-00472-x (PMC9066965; doi:10.1186/s10020-022-00472-x)
Supplement: Supplementary file 3 — Additional file 3: Table S1. Individual clinical baseline characteristics of patients with preeclampsia. Table S2. Selected individual baseline characteristics of pregnant controls. [file 10020_2022_472_MOESM3_ESM.pdf]

Supplemental Table 1: Individual clinical baseline characteristics of patients with preeclampsia.

No.	Age	GA	Blood pressure syst./ diast. (mmHg)	Proteinuria	Platelets *1000/µl	Transaminases	sFlt1/ PlGF	Annexin V	CD62P
1	38	34	150/ 95	+	193	-	154	N/A	N/A
2*	33	37	210/ 120	++	144	-	73	+	-
3	43	29	150/ 102	-	131	+	334	N/A	N/A
4	28	37	180/ 120	++	117	-	209	N/A	N/A
5	31	38	115/ 71	+	106	++	121	+	-
6	33	37	104/ 73	-	98	++	62	+	+
7	23	33	150/ 100	+	191	-	147	-	-
8	39	36	160/ 90	++	140	++	71	+	-
9	24	29	147/ 90	+	238	-	609	+	+
10	30	35	150/ 100	+	113	-	82	+	-
11	32	34	170/ 95	+	176	+	123	+	-
12	35	37	155/ 97	-	127	+	131	-	-
13	30	37	153/ 92	+	169	-	138	+	+
14	35	26	180/ 115	++	223	-	612	-	-
15	31	35	172/ 117	++	163	-	182	-	-
16	27	28	168/ 112	++	180	++	502	+	+

Proteinuria: (+) – proteinuria of 300 mg/gCreatine to 2000 mg/gCrea;
(++) – above 2000 mg/gCrea.
Transaminases: (+) – 2 to 3-fold increase; (++) – above 3-fold increase.
red: severe preeclampsia; black – non-severe PE. GA – gestational age.
* severe headache/neurological deficits as severe PE criteria.
Annexin V/CD62 (P-Selectin): (+) – surface overexpression versus pregnant control;
(-) no overexpression. N/A – no available data.

Supplemental Table 2: Selected individual baseline characteristics of pregnant controls.

**No.**	**GA**	**Age**	**Blood pressure systolic/ diastolic (mmHg)**	**Platelets *1000/µl**
1	42	39	102/ 71	127
2	39	39	120/ 73	202
3	34	34	102/ 67	181
4	31	40	111/ 68	193
5	43	38	129/ 70	136
6	33	33	114/ 70	226
7	26	24	116/ 77	165
8	32	40	118/ 78	126
9	40	39	124/ 75	195
10	37	36	103/ 72	245
11	30	36	115/ 78	203
12	29	40	126/ 76	153
13	26	39	121/ 70	209
14	19	39	126/ 76	268
15	34	32	124/ 75	168
16	37	41	120/ 77	189

GA – gestational age.
